# Measurement of Microcystin and Nodularin Activity in Human Urine by Immunocapture-Protein Phosphatase 2A Assay

**DOI:** 10.3390/toxins11120729

**Published:** 2019-12-13

**Authors:** Rebekah E. Wharton, Brady R. Cunningham, Adam M. Schaefer, Sophia M. Guldberg, Elizabeth I. Hamelin, Rudolph C. Johnson

**Affiliations:** 1 Division of Laboratory Sciences, National Center for Environmental Health, Centers for Disease Control and Prevention, Atlanta, GA 30341, USA; nyj6@cdc.gov (B.R.C.); eph3@cdc.gov (E.I.H.); rmj6@cdc.gov (R.C.J.); 2 Harbor Branch Oceanographic Institute, Florida Atlantic University, Ft. Pierce, FL 34946, USA; Aschaef3@fau.edu; 3 Oak Ridge Institute for Science, Centers for Disease Control and Prevention, Atlanta, GA 30341, USA; sophia.guldberg@gmail.com

**Keywords:** microcystins, nodularin, protein phosphatase 2A, immunocapture, harmful algal bloom

## Abstract

Microcystins (MC) and nodularin (NOD) are toxins released by cyanobacteria during harmful algal blooms. They are potent inhibitors of protein phosphatases 1 and 2A (PP1 and PP2A) and cause a variety of adverse symptoms in humans and animals if ingested. More than 250 chemically diverse congeners of MCs have been identified, but certified reference materials are only available for a few. A diagnostic test that does not require each reference material for detection is necessary to identify human exposures. To address this need, our lab has developed a method that uses an antibody to specifically isolate MCs and NOD from urine prior to detection via a commercially available PP2A kit. This assay quantitates the summed inhibitory activity of nearly all MCs and NOD on PP2A relative to a common MC congener, microcystin-LR (MC-LR). The quantitation range for MC-LR using this method is from 0.050–0.500 ng/mL. No background responses were detected in a convenience set of 50 individual urines. Interday and intraday % accuracies ranged from 94%–118% and relative standard deviations were 15% or less, meeting FDA guidelines for receptor binding assays. The assay detected low levels of MCs in urines from three individuals living in close proximity to harmful algal blooms (HABs) in Florida.

## 1. Introduction

**Figure 1:**
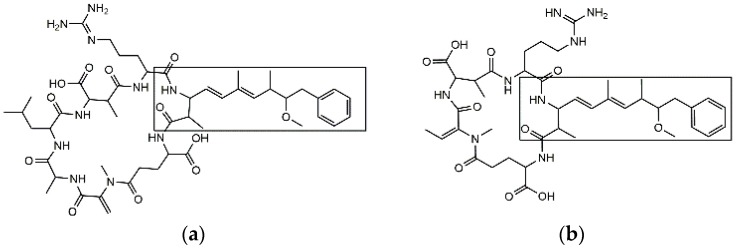
The structure of microcystin-LR (MC-LR) (**a**) and nodularin (NOD) (**b**). The boxed portion denotes the adda moiety which is conserved amongst microcystin (MC) congeners and NOD.

Cyanobacteria are ubiquitous photosynthetic bacteria commonly found in freshwater systems worldwide. Eutrophication stimulates cyanobacterial proliferation which can result in the formation of harmful algal blooms (HABs). During HABs, species of cyanobacteria release toxic peptides, including microcystins (MCs) and nodularin (NOD), into waterways [1]. Only one form of NOD exists, but nearly 250 MC congeners have been reported [2]. While significant structural diversity exists among MC congeners and NOD, most contain a unique amino acid, 3-amino-9-methoxy-2,6,8-trimethyl-10-phenyldeca-4,6-dienoic acid (adda). The structures of a common and highly toxic MC congener, microcystin-LR (MC-LR), and NOD are shown below (Figure 1).

Humans can become exposed to MCs by a variety of routes including ingestion, direct skin contact, haemodialysis, or inhalation [3]. MCs and NOD are potent inhibitors of protein phosphatases 1 and 2A (PP1 and PP2A). Human exposures to these toxins can lead to a variety of symptoms ranging from gastroenteritis, nausea, allergic reactions, and skin rashes in mild cases to hepatic injury and hemorrhage in more severe cases [4]. MCs have also been linked to tumor progression and are harmful to renal, immune, and reproductive systems [5,6,7,8]. Due to the potential harm to humans, the World Health Organization (WHO) has set the upper provisional guideline limit of 1 ng/mL for MC-LR in their *Guidelines for Drinking Water Quality* [9].

The most common techniques used for detection of MCs, include mass spectrometry [10,11,12,13,14,15], enzyme-linked immunosorbent assay (ELISA) [16,17,18], liquid chromatography photodiode array detection (LC-PDA) [19,20], protein phosphatase inhibition assay (PPIA) [21,22,23,24], and the mouse bioassay [25]. While each detection technique has unique advantages and disadvantages, only the PPIA can provide information on the biological activity of MCs and NOD in samples without the use of live animals. Historically, the colorimetric PPIA’s usefulness as a screening tool has been limited by its inability to distinguish between different classes of PP2A inhibitors such as MCs, okadaic acid, and calyculin A, and its sensitivity. Our lab has improved the sensitivity and specificity of the traditional PPIA assay by incorporating an immunocapture step. The developed immunocapture protein phosphatase inhibition assay (IC-PPIA) uses an adda-specific antibody to capture and 10-fold concentrate only MCs and NOD from urine prior to PPIA toxicity measurements relative to MC-LR. This assay can be used as a diagnostic screening tool to monitor low-level human exposures to MCs and NOD.

## 2. Results

### 2.1. Method Optimization

**Figure 2:**
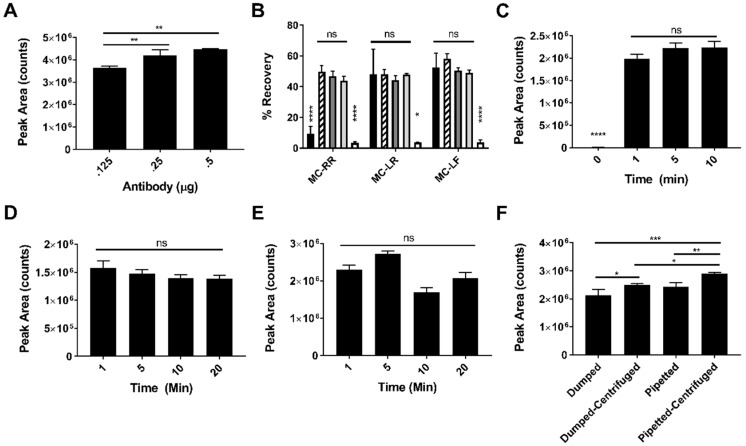
MC-LR immunocapture (IC) optimization. All optimization experiments were performed using 500 µL of 1 ng/mL singly-charged (MC-LR) congeners in pooled urine. In panel B, 500 µL of 1 ng/mL doubly-charged (MC-RR) and uncharged (MC-LF) congeners in pooled urine were also used. MC antibody titration to optimize capture of MC-LR from pooled urine (*n* = 3) (**A**). Selection of optimal elution buffer for IC of three MC congeners. Black bar (100% ACN/0.5% FA), striped bar (70% ACN/30% water/0.5% FA), dark gray bar (50% ACN/50% water/0.5% FA), light gray bar (30% ACN/70% water/0.5% FA), white bar (100% water/0.5% FA), (*n* = 3) (**B**). Capture time optimization for antibody conjugation to magnetic beads (*n* = 3) (**C**). Capture time optimization of MC-LR from pooled urine (*n* = 3) (**D**). Time optimization for eluting MC-LR from magnetic beads (*n* = 3) (**E**). Optimal conditions for removing supernatants from beads (*n* = 3) (**F**). Significance was determined by one-way ANOVA and Tukey’s multiple comparisons post-test. * *p* ≤ 0.05, ** *p* ≤ 0.01, *** *p* ≤ 0.0005, **** *p* ≤ 0.0001, ns = not significant. Error bars represent the standard deviation of replicate samples. % Recovery = peak area of pre-spike sample/peak area of post-spike sample × 100%.

Our lab previously described a method for detection of MC-LR in human urine by immunocapture (IC) liquid chromatography tandem mass spectrometry [26]. The IC protocol from this method was adapted for the IC-PPIA method described here for detection of all MCs and NOD by reoptimizing reagent amounts for this method’s detection range, addition of a buffering step for compatibility with PP2A activity measurement, and adjusting sample processing for improved recovery. First, the amount of antibody necessary for IC was optimized. Briefly, biotinylated MCs antibodies were coupled to streptavidin magnetic beads at the saturation ratio provided by the bead manufacturer. Various conjugated bead volumes corresponding to 0.125, 0.250, and 0.500 µg MC antibody were incubated with 1 ng/mL MC-LR (the most concentrated calibrator). Although no significant differences in peak area were observed between 0.25 and 0.50 µg antibody samples, residual MC-LR was detected in the urine of the 0.25 µg sample after IC (data not shown), so 0.5 µg antibody was selected as the optimal amount (Figure 2A).

The elution buffer composition was optimized next. Representative doubly-charged (MC-RR), singly-charged (MC-LR), and uncharged (MC-LF) congeners were selected for analysis to encompass the structural diversity observed among MCs. Each incubation step was performed for 20 min to ensure sufficient binding or elution time was allowed. Elution buffers comprised of 0.5% formic acid water and varying concentrations of acetonitrile and water were tested to determine which yielded the best recoveries for each congener. Previous studies performed by our lab concluded formic acid was necessary for effective elution of MCs from the antibody [26]. Elution buffers containing mixtures of water and acetonitrile yielded recoveries around 50% for all congeners tested, whereas elution buffers containing only water yielded recoveries around 5%. Elution buffers comprised of only acetonitrile yielded low recovery of MC-RR (~10%), but were similar to the water/acetonitrile mixtures for the other congeners tested (~50%). Because 70% water/30% acetonitrile/0.5% formic acid would be most compatible with the downstream PPIA, it was selected as the optimal buffer for the assay (Figure 2B). Percent recoveries were calculated by comparing peak areas of MCs spiked in pooled urine prior to IC with blank urine spiked with each MC post-IC.

The minimum time required for the antibody to bind to the magnetic bead was determined by varying the incubation times. The incubation times tested were 0, 1, 5, and 10 min. The 0 min time reflects non-specific binding levels of MC-LR to the magnetic beads with no antibody present. We found 1 min to be adequate to conjugate the antibody to the magnetic bead since there was no significant difference in peak area at later time points. No residual MC-LR was detected in the urine after IC (data not shown). Non-specific binding of MC-LR to the beads was minimal (Figure 2C).

Capture of MC-LR onto the antibody-conjugated beads was optimized by incubating 500 µL of 1 ng/mL MC-LR in urine for varying times. Incubation times of 1, 5, 10, and 20 min were tested. The 1 min incubation was sufficient for capture since there was no significant difference in peak area at later time points (Figure 2D) and no residual MC-LR was observed in the urine after IC (data not shown).

The elution of the MC-LR from the antibody-bead complex was optimized by varying elution times. The incubation times of 1, 5, 10, and 20 min were tested. The 1 min incubation time was adequate to elute MC-LR from the antibody since no significant increases in peak area were observed between 1 and 20 min incubations (Figure 2E).

Centrifuging MC-LR spiked urine samples to remove particulates prior to IC and pipetting off urine supernatants after IC instead of dumping and blotting were evaluated to identify the optimal way to process samples. The highest peak areas resulted when urine samples were both centrifuged prior to IC and urine supernatants removed by pipetting after IC (Figure 2F)

### 2.2. Recovery of MC-RR, MC-LR and MC-LF after IC

**Figure 3:**
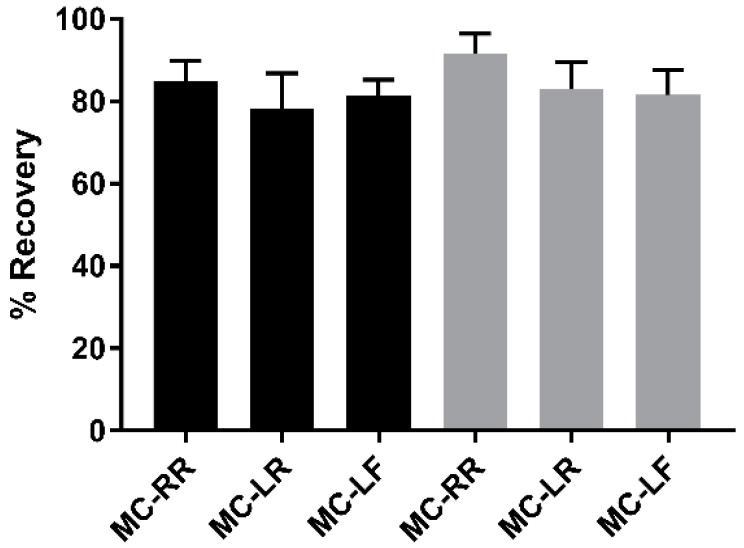
Method recovery of pooled urine samples spiked with 0.250 (black bars) or 0.080 ng/mL (gray bars) of MC-RR, MC-LR, or MC-LF prior to IC compared to post-spike controls. % Recovery = concentration of pre-spike sample/concentration of post-spike sample × 100%. Statistical significance of data was determined by one-way ANOVA and Tukey’s multiple comparisons post-test (*n* = 3).

Because the IC elution buffer is highly acidic, it needed to be buffered to make it compatible with PP2A activity in the downstream PPIA. Ammonium bicarbonate was selected for this purpose since it has a neutral buffering range (pH 6.6–8.6). The coupled IC-PPIA was used to evaluate the recovery of three MC congeners. Final recoveries were determined by comparing the interpolated concentrations of spiked urine containing 0.250 or 0.080 ng/mL of MC-LR, MC-RR, and MC-LF prior to IC with a post-spike control of blank urine fortified with 2.50 or 0.800 ng/mL after IC (Figure 3). Percent recoveries were comparable between congeners and spiked concentration levels. The average % recovery amongst congeners was 83.4%.

### 2.3. Specificity

**Figure 4:**
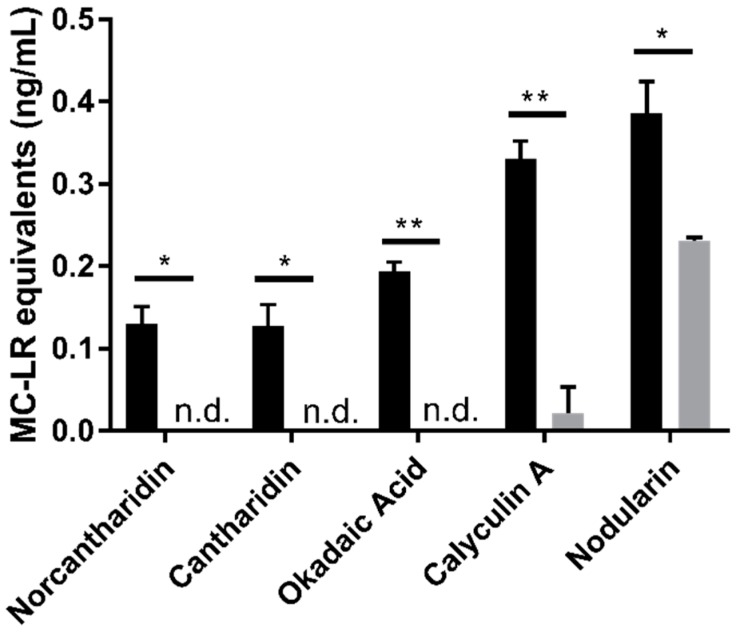
Method interferences from known PP2A inhibitors. Pooled urine spiked with 0.500 ng/mL of PP2A inhibitors prior to IC (gray bars) were compared to 5.00 ng/mL post-spike controls (black bars). Significance was determined by unpaired *t*-test. * *p* ≤ 0.05, *** *p* ≤ 0.005, **** *p* ≤ 0.0001, n.d. = not detected. Error bars represent the standard deviation of duplicate samples.

PP2A is inhibited by other small molecules than MCs and NOD, including norcantharidin, cantharidin, okadaic acid, and calyculin A. Cantharidin, and its demethylated derivative, norcantharidin, are antitumor drugs that have been evaluated in numerous clinical trials [27]. Okadaic acid and calyculin A are naturally occurring marine toxins [28,29]. The method specificity for MCs was tested by spiking each PP2A inhibitor into pooled urine at concentrations ranging from 0.050–5.00 ng/mL prior to IC. The resulting signals were compared to blank pooled urine spiked with each compound after IC (post-spike control). Recovery trends were the same for all inhibitor concentrations tested. All compounds tested, except for NOD, had negligible responses after IC at all concentrations tested. The recovery of NOD was expected since the IC antibody recognizes the adda moiety, which is conserved between MCs and NOD. The recovery of NOD in this experiment was 60.0% (Figure 4).

### 2.4. Validation of MC-LR

**Figure 5:**
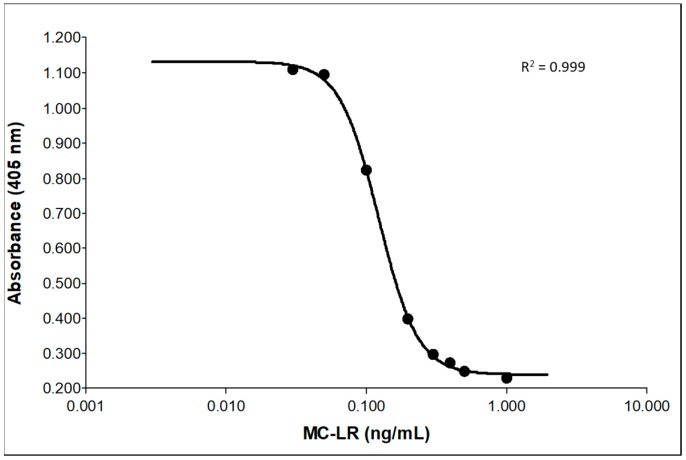
Representative calibration curve of MC-LR from validation. Points at 1.00 and 0.030 ng/mL are curve anchors.

To validate the IC-PPIA, 20 calibration curves and QC materials spiked at 0.250 (QH) and 0.080 ng/mL (QL) were evaluated by two analysts over the course of five weeks. Two kit lots were used during the validation. Interday percent accuracies varied between 94.2%–118% and relative standard deviations (RSDs) ranged from 2.16–15.0 (Table 1). Intraday percent accuracies were between 100% and 113% with RSDs from 10.5–12.7 (Table 1). Optimal four parameter fit curves were facilitated by the use of anchor points at 1.00 and 0.030 ng/mL MC-LR. Use of the anchor points significantly improved accuracy and precision of the 0.500 ng/mL calibrator and prevented false positives, but did not meet quantitation criteria for accuracy and RSD. The method quantitation range was from 0.050–0.500 ng/mL and a representative calibration curve including anchor points is shown in Figure 5. The method limit of detection, calculated using the Clinical Laboratory and Standards Institute (CLSI) EP17 method described previously, was 0.0283 ng/mL [30]. A convenience set of 50 individual urines with no known exposure to MCs were evaluated for responses using the method. Unexposed samples tested had no detectable levels of MCs (data not shown). Overall, these data meet current U.S. Food and Drug Administration’s guidelines for ligand binding assays [31].

**Figure 6:**
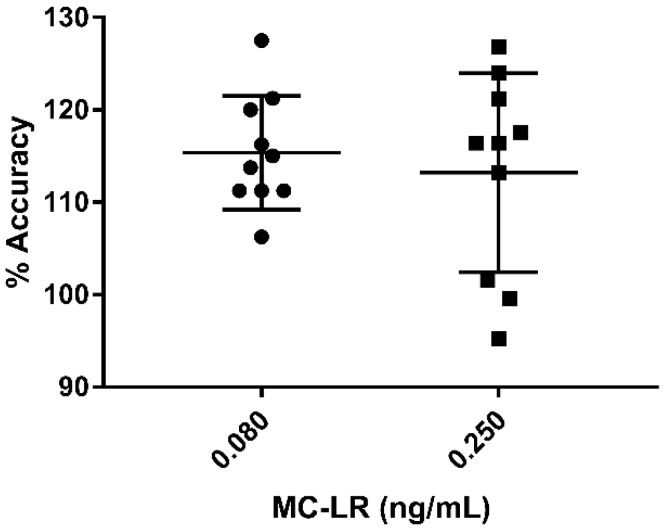
Individual urine samples were spiked with 0.080 and 0.250 ng/mL MC-LR and evaluated for % accuracy and precision (*n* = 10 for each spike level). Error bars represent the standard deviation.

Ten individual urines from a convenience set were spiked with 0.080 or 0.250 ng/mL of MC-LR. The average intraday % accuracy for the 0.080 ng/mL spiked samples was 115% and 113% for 0.250 ng/mL spiked samples. The intraday RSD was 5.33 for the 0.080 ng/mL samples and 9.53 for the 0.250 ng/mL spiked samples (Figure 6).

### 2.5. Analysis of Urine Samples from Florida Residents

During the summer of 2018, south Florida experienced a significant cyanobacteria algal bloom. In order to assess human exposure to MCs, urine, nasal swabs, and blood were collected from residents of St. Lucie, Indian River, Palm Beach, and Martin counties as a part of a cross-sectional exposure study. A comprehensive questionnaire that included questions on potential routes of exposure to the blooms, fish consumption, and demographic data was administered at the time of sample collection. A total of 121 individuals were recruited and sampled from August to November 2018.

Concurrent with the human subject recruitment, multiple agencies collected water and algae samples for bloom and toxin surveillance. Between August and November surface water concentrations of MCs from the counties listed above ranged from below detection to 110 µg/L [32]. More specific geographical and spatial-temporal environmental sampling and human exposure data are currently being analyzed and will be reported elsewhere.

Of the 121 individuals recruited, 86 provided urine samples with sufficient volume and quality for analysis. Three of the 86 urine specimens analyzed by the IC-PPIA method described in this paper yielded positive results. The concentrations of these samples were 0.055, 0.089, and 0.052 ng/mL MC-LR equivalents. Two of the participants reported living directly on an impacted waterway and the third managed a marina.

## 3. Discussion

Our lab has developed an IC-PPIA to detect total MCs and NOD in human urine. This method can quantitate a common and highly toxic variant of MC, MC-LR, from 0.050–0.500 ng/mL with high accuracy and precision. Other MC congeners and NOD can also be measured in equivalents relative to MC-LR. The use of an adda-specific antibody for sample cleanup effectively captures MCs and NOD while removing other PP2A inhibitors and most matrix interferences from samples. The high specificity of the antibody used for sample cleanup in addition to the low pH elution buffer (pH ~2) make substrate dephosphorylation from endogenous urine contaminants unlikely. Furthermore, individual urines spiked with MC-LR were quantitated and average values fell within 15% of theoretical values with RSDs less than 10, although they tended to bias high. This is likely due to a slight matrix effect of the individual urines compared to the pooled urine used in validation. IC also provides a simple method to ten-fold concentrate samples, allowing a very low method limit of detection. The method is compatible with high-throughput sample analysis and automation since all steps are performed in a 96-well plate. All reagents for this method can be obtained commercially and since only a small amount is required for each sample, the method is inexpensive.

The relevance of this method to detect low-dose human exposures to MCs was demonstrated by analysis of urines from persons living or working in close proximity to nearby algal blooms in Florida. MC-LR equivalent levels in these specimens ranged from 0.052–0.089 ng/mL. Questionnaire responses from study participants with positive MC test results indicate they were likely exposed via inhalation, as they did not report having direct contact with contaminated water. Future studies examining potential long-term health effects associated with MCs in populations living in close proximity to HABs would be interesting in light of this information.

MC-LR has previously been detected in the urine and plasma of mice injected intravenously or intraperitoneally with the toxin, despite rapid clearance [33]. Additional studies performed in mouse and rat models have detected cysteine and glutathione MC conjugates in liver cytosols, but they have not yet been reported in urine [34]. If present in urine, these metabolites would likely be detected by the developed assay since the site of glutathione and cysteine conjugation is distinct from the adda group recognized during IC and these conjugates have previously been reported to inhibit PP2A with only slightly less activity than MC-LR [35]. Detection of these metabolites was not directly tested in this study since MC glutathione and cysteine conjugates are not commercially available.

This method has several advantages to other detection techniques currently used for detection of MCs and NOD. First, the method has two layers of specificity instead of one in a traditional competitive ELISA. Because MCs and NOD must be recognized by a specific antibody in addition to inhibiting PP2A, the incidence of false positives and false negatives should be reduced compared to ELISA. Similar to the mouse bioassay, this method is able to provide biological activity related to MC and NOD exposures, but is less expensive and does not require the use of live animals. Mass spectrometry-based methods are important in identifying individual congeners of MCs, but their application in detecting low-level human exposures may be limited by sensitivity based on the levels measured by the IC-PPIA assay described here [36,37]. Since multiple MC congeners are typically present in water samples during cyanobacterial blooms, a method capable of measuring all MCs is advantageous so low-level exposures will not be missed [38,39]. Finally, because all measurements are relative to MC-LR, only one certified reference material is necessary for analysis unlike other techniques such as tandem mass spectrometry which rely on individual reference materials for each congener.

In conclusion, this work describes the development and application of an IC-PPIA for detection of nearly all MCs and NOD in human urine. Ten-fold sample concentration in this method make it sensitive enough to detect even low-level human inhalation exposures to MCs and NOD. This method could complement water monitoring programs by identifying human exposures to MCs and NOD at the time of HABs and may assist in elucidating health effects associated with these toxins in the future.

## 4. Materials and Methods

### 4.1. Materials

MC-LR and MC-RR certified standards, the anti-adda antibody (AD4G2), and the assay kit for detection of MCs/NOD in water by PP2A were purchased from Abraxis, Inc (Warminster, PA, USA). MC-LF, NOD, cantharidin, okadaic acid, and calyculin A standard were purchased from Enzo (Farmingdale, NY, USA). Norcantharidin and phosphate buffered saline, pH 7.4 with Tween 20 (PBS-T) were obtained from Sigma-Aldrich (St. Louis, MO, USA). All MCs and other PP2A inhibitors were stored at −20 °C. The antibody was stored at 4 °C. Acetonitrile was from Pharmaco (Dawsonville, GA, USA). Methanol and formic acid were obtained from Fisher Scientific (Waltham, MA, USA). Life Technologies Corporation (Grand Island, NY, USA) supplied the Dynabeads MyOne Streptavidin T1, Zebaspin 7K molecular weight cut off, 0.5 mL desalting columns, and EZ-link NHS-PEG4-biotin. The Thermomixer C, Protein LoBind 2 mL microcentrifuge tubes, Protein LoBind 1 mL deep well plates, and TwinTec Lo-Bind PCR plates were purchased from Eppendorf (Hauppage, NY, USA). V&P Scientific (San Diego, CA, USA) supplied the 96-well plate magnet (part number VP771HH-MC). The microcentrifuge tube magnet was purchased from Invitrogen (Waltham, MA, USA).

### 4.2. Biological Specimens

Individual and pooled urine samples used during method validation were purchased from Tennessee Blood Services (Memphis, TN, USA). These specimens were from anonymous, random individuals and thus this work did not meet the definition of human subjects research as specified in 45-CFR 46.102 (f). Urines from the Florida study were self-collected by participants into sterile collection cups and stored at −80 °C until shipment to the Florida Atlantic University laboratory. All human subject research was conducted in accordance with the Declaration of Helsinki, and was approved and monitored by the Florida Atlantic University Institutional review board (#929397-1). CDC’s role in this human subject research does not require HHS human subjects review as they served as a technical assist and were not engaged.

### 4.3. Preparation of Anchors, Calibrators, and Quality Control Samples

All anchors, calibrators, and quality control samples were prepared and stored in Eppendorf Protein LoBind microcentrifuge tubes. LoBind tubes have been shown to reduce sample loss since MCs stick and bind to polypropylene [40]. A 14.6 µg/mL stock of MC-LR in methanol was diluted to make the 50.0 ng/mL working stock in pooled urine. The working stock was diluted in pooled urine to create the anchors, 1.00 and 0.0300 ng/mL, the calibrators, 0.500, 0.400, 0.300, 0.200, 0.100, and 0.0500 ng/mL MC-LR, and quality control (QC) samples, 0.250 (QH) and 0.0800 (QL) ng/mL MC-LR. Calibrators were divided into single use aliquots and stored at −20 °C until needed.

### 4.4. Mass Spectrometry Conditions

Peak area was measured using a Thermo Scientific Q Exactive Hybrid Quadrupole-Orbitrap mass spectrometer (Waltham, MA, USA) with a Heated Electrospray Ionization (HESI-II) probe operating in positive ion mode. Scans were performed using Full MS/AIF with MC-LR input at 995.55640 *m*/*z* in the inclusion list. Sheath gas, 45 psi; Auxiliary gas, 10 psi; sweep gas, 1 psi; ion spray voltage, 6500 V; S-lens RF level, 100.0; source temperature, 320 °C; capillary temperature, 450 °C. Full MS resolution, 70,000; AGC target; 3e6, Maximum IT, 50 ms; scan range, 400 to 1200 *m*/*z*. AIF resolution, 35,000; AGC target, 3e6; Maximum IT, 500 ms; N(CE)/stepped, 35, 67; scan range, 80 to 600 *m*/*z*.

### 4.5. Liquid Chromatography Conditions

MC-LR was resolved on an Agilent 1290 liquid chromatograph (Santa Clara, CA, USA) using an Acquity UPLC BEH C18 column (130 Å, 1.7 µm, 2.1 × 50 mm, Waters, Milford, MA), maintained at 20 °C. Mobile phase (A) was comprised of 0.1% (*v*/*v*) formic acid in DI water and (B) was comprised of 70:30:0.1% (*v*/*v*) acetonitrile:methanol:formic acid. The elution pump program was as follows: flow rate of 500 µL/min with 30% B initial conditions rising to 95% B at 6.0 min, a quick decrease back to 30% B at 6.1 min and maintained until 8.0 min. Retention time and peak area were reproducible suggesting that the 0.1 min transition from high to low organic was sufficient to condition the column.

### 4.6. MC IC Protocol

The MC antibody was biotinylated at a 30 biotin:1 antibody molar coupling ratio using an EZ-link NHS-PEG4-biotin kit per the manufacturer’s instructions. After biotinylation, unbound biotin was removed by passing the sample through a Zebaspin desalting column. Biotinylated antibody was incubated with streptavidin magnetic beads at a ratio of 0.5 µg antibody:2.5 µL beads/sample in a protein LoBind 2 mL microcentrifuge tube. The sample was mixed for 30 s at 800 rpm followed by 30 s without mixing at 25 °C on a Thermomixer C. The conjugated magnetic beads were washed two times and reconstituted in phosphate buffered saline containing Tween 20 (PBS-T), then 25 µL aliquots were pipetted into a 1 mL protein LoBind deep well plate. The anchors, calibrators, and QCs were centrifuged at 3000 rpm for 3 min to pellet any particulates in the urine. Five hundred microliters of each urine supernatant were then incubated with the antibody-conjugated beads for 1 min at 25 °C on a Thermomixer C mixing at 1850 rpm. The beads were separated from the sample using the 96-well magnet, and the supernatant was removed by pipette. Next, 42.5 µL of elution buffer (70% DI water:30% acetonitrile w/0.5% formic acid) was added to the beads, and the plate was vortexed for 1 min on a Thermomixer C at 25 °C at 1400 rpm. After elution, the beads were separated on the 96-well plate magnet, and the supernatant was transferred to the PP2A kit plate. We added 7.5 L of 1 M ammonium bicarbonate to each sample to adjust the pH to 6–8 and bring the final sample volume to 50 µL. The pH adjustment was necessary to ensure proper enzyme activity in the PPIA.

### 4.7. Protein Phosphatase Inhibition Assay

Before beginning the assay, the PP2A assay kit was brought to room temperature. The PP2A was hydrated in kit dilution buffer by rotating for 1 h at 25 °C on a rotating mixer. After PP2A hydration, 70 µL of the phosphatase solution were added to each sample followed by 90 µL of the kit chromogenic substrate. The wells were covered with adhesive film and incubated on a Thermomixer C for 30 min at 37 °C. After incubation, 70 µL of stop solution was added to each sample, and the absorbance was read at 405 nm on a BioTek Powerwave HT microplate spectrophotometer (Winooski, VT, USA). Spectrometer calibration was verified daily before sample analysis.

### 4.8. Data Analysis

Absorbance data were analyzed using Gen5 v. 2.04 (BioTek, Winooski, VT, USA) software. All calibrators were fit to a 4-parameter curve fitting. Prism verison 7 was used for all statistical analyses.

## Figures and Tables

**Table 1:** Validation results. Representative interday (*n* = 20) and intraday (*n* = 5) RSDs and % accuracies of calibrators and QC samples.

	Sample (ng/mL)	Average (ng/mL)	% Accuracy	RSD
**Interday**	0.500	0.471	94.2	9.80
	0.400	0.376	94.0	10.1
	0.250 (QH)	0.295	118	7.82
	0.200	0.203	102	3.91
	0.100	0.100	100	2.16
	0.080 (QL)	0.086	108	8.66
	0.050	0.053	106	15.0
**Intraday**	0.250 (QH)	0.283	113	12.7
	0.080 (QL)	0.080	100	10.5
